# Comparison of clinical results of two pharmaceutical products of riboflavin in corneal collagen cross-linking for keratoconus

**DOI:** 10.1186/2008-2231-22-37

**Published:** 2014-04-08

**Authors:** Hassan Hashemi, Mohammad Amin Seyedian, Mohammad Miraftab, Hooman Bahrmandy, Araz Sabzevari, Soheila Asgari

**Affiliations:** 1 Noor Ophthalmology Research Center, Noor Eye Hospital, No. 96 Esfandiar Blvd., Vali’asr Ave., Tehran, Iran; 2 Faculty of Pharmacy, Tehran University of Medical Sciences, Tehran, Iran

**Keywords:** Riboflavin, Pharmaceutical product, Sina Darou, Cross linking, Keratoconus, Clinical trial

## Abstract

**Background:**

To compare the 6-month results of two formulations of Riboflavin provided by Sina Darou, Iran, and Uznach, Switzerland, in corneal collagen cross-linking (CXL) for keratoconus patients.

**Findings:**

Considering the results of the previous study about the similarity of the formulations and the active ingredients of the two types of Riboflavin, they were used in the CXL procedure of 60 keratoconic eyes (30 eyes in each group). After 6 months, the mean improvement of UCVA (0.239), BCVA (0.707), and MRSE (0.513) did not differ significantly between the two groups. The mean decrease in max- K (0.731), mean- K (0.264), central corneal thickness (0.759), and Q-value (0.669) did not show any significant difference between the two groups. The two groups had no significant difference in endothelial cell count decrease (0.229). The Sina Darou formulation decreased corneal hysteresis more than the Swiss formulation (P = 0.057) but there were no significant differences in the mean decrease of corneal resistance factor between the two groups (P = 0.117).

**Conclusions:**

Based on the early results, the results of visual acuity, refraction, and corneal topography using Sina Darou and Uznach formulations of Riboflavin showed that both were effective in CXL. However, considering the relatively significant difference in corneal hysteresis changes between the two groups, this study will continue to report the long-term results.

## Background

Collagen cross linking (CXL) with Riboflavin has shown desirable effects on the arrest of keratoconus [1-3]. In this procedure, riboflavin produces free radicals under the effect of UV. These radicals create new covalent bonds in the stroma which strengthen the corneal tissue [1]. Riboflavin enhances UVA absorbance as a photosensitizer [4] and reduces cellular damage [5]. Therefore, the use of riboflavin in CXL is of extreme importance. In a primary study, we showed that the formulation and the amount of the active ingredient of riboflavin produced in Sina Darou, Iran, were similar to riboflavin produced in Uznach, Switzerland. Fluorometry and high performance liquid chromatography were used to compare the amount of the active ingredient of the two products. After calculating the area under curve of the absorption rate of the active ingredient of the two products, statistical analysis showed no significant difference between them. In the present study, the efficacy of the two pharmaceutical products was compared in order to recommend the use of the Iranian product, which is more available and has lower costs, instead of the Swiss product.

## Finding

### Methods

**Figure 1:**
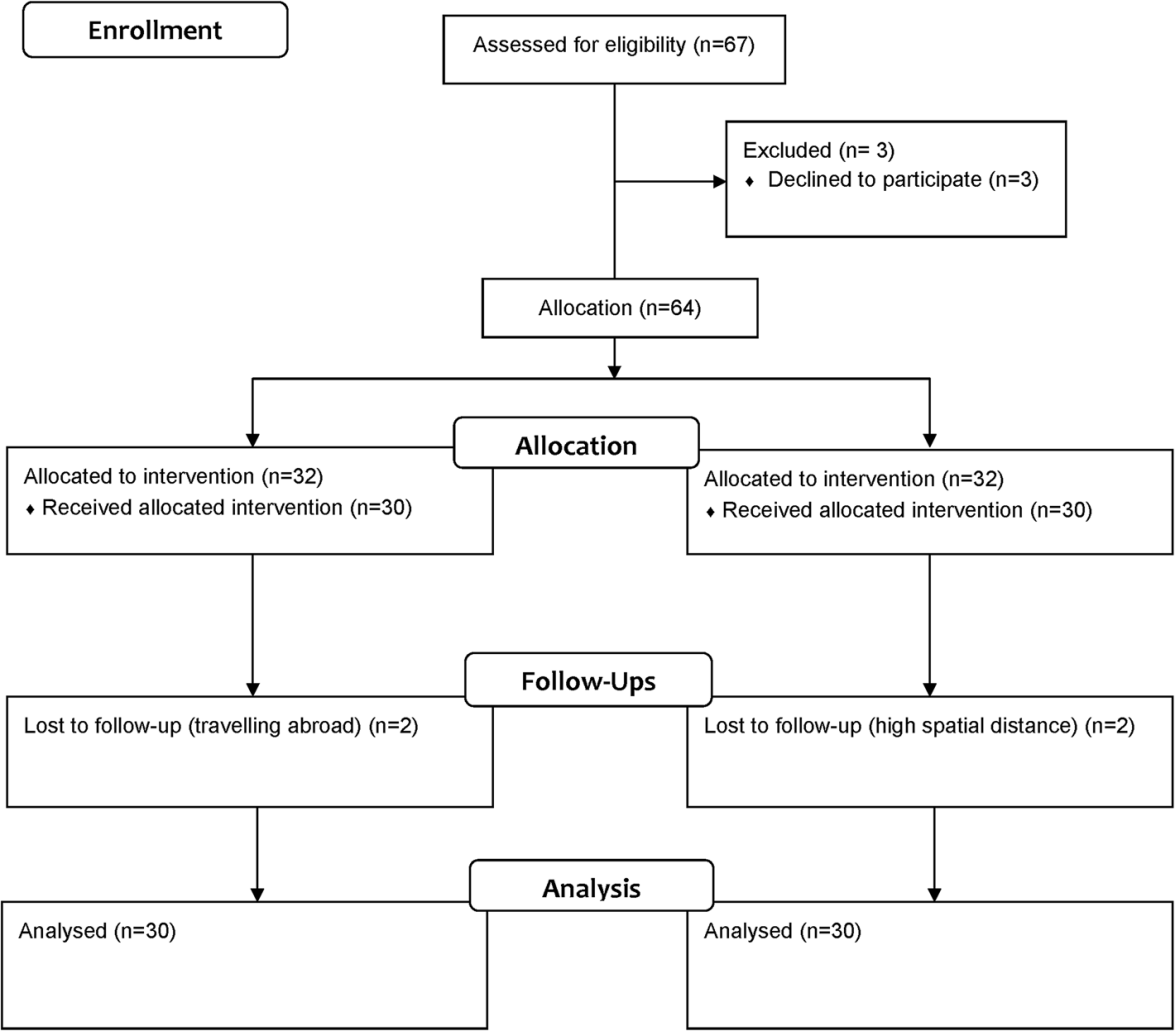
Flow Diagram of the passage of participants.

In this parallel clinical trial, 60 eyes of 60 keratoconus patients that received CXL were compared in two groups. The flowchart of the passage of participants is shown in Figure 1. Iranian riboflavin 0.1% (Sina Darou, Iran) was used during CXL in group A and Swiss riboflavin 0.1% (Streuli Pharmaceuticals, Uznach, Switzerland) was used in group B. The patients were allocated to the groups consecutively. The inclusion criteria were the clinical and paraclinical diagnosis of progressive keratoconus, age 15–35 years, keratometry less than 55 D, and a central corneal thickness (CCT) more than 400 micron. Patients with other ocular diseases or a history of ocular surgery were excluded from the study. Participants discontinued the use of the hard and soft contact lens 3 weeks and 3 days prior to the surgery, respectively.

At first, a written informed consent was obtained from each participant. Noor review board approved the study. Iranian Registry of Clinical Trials also approved the study (registration number: IRCT201212034333N2).

The method of the surgery has been already reported [3]. After local anesthesia, the epithelium of the central 7 mm of the cornea was removed in 3–4 vertical strips that measured about 2 mm in width sparing a strip measuring approximately 1 mm. An epithelial strip was also horizontally removed from the lower one third of the cornea. Then, Iranian or Swiss riboflavin 0.1% drops in dextran 20% were instilled on the cornea every 3 minutes for 30 minutes in the intervention and control group, respectively. After the saturation of riboflavin in the anterior chamber, irradiation (wavelength 370 nm, power 3 mW/cm^2^) was started at 5 cm using UVX system (IROC, Zürich, Switzerland). During 30 minutes of irradiation, riboflavin instillation was repeated every three minutes. Then, the corneal surface was irrigated with sterile balanced saline solution, a soft bandage contact lens (Night & Day, Ciba Vision, Duluth, GA) was placed on it, and chloramphenicol 0.5% (Sina Darou, Iran) was administered. Post surgical regimen included chloramphenicol 0.5% four times daily, betamethasone 0.1% (Sina Darou, Iran), and preservative free artificial tears (Hypromelose, preservative free) as required. The patients were examined on days 1 and 3 after surgery, and the lens was removed if healing was observed. After removing the lens, chloramphenicol was discontinued while betamethasone twice daily was continued for another week. If epithelial healing was not observed, daily visits continued until the epithelium healed completely. No complications were noticed during or after surgery. Corneal haze related to the surgery was completely removed before the third month.

The patients received ophthalmic examination and paraclinical evaluation before the procedure and 1, 3, and 6 months after it. Paraclinical tests included uncorrected visual acuity (UCVA) and best spectacle corrected visual acuity (BSCVA) using a Snellen chart, and manifest refraction spherical equivalent (MRSE) using an Auto refractometer (Topcon KR-8800, Japan). We used Pentacam (Oculus Optikgerate GmbH, Germany) to evaluate corneal topographic indices, an ocular response analyzer (ORA; Reichert Ophthalmic Instruments, Buffalo, USA) to assess corneal biomechanical properties, and a non-contact specular microscope (Konan Medical, Hyogo, Japan) to investigate endothelial cell count (ECC). The corneal biomechanical properties and ECC were evaluated twice, once before the surgery and once six months after the procedure.

We used repeated measure analysis of variance to evaluate the trend of the changes in each group and between the two groups. The results are shown as mean ± SD. The level of significance was set at 0.05.

## Results

Sixty eyes of 60 patients (60% male) with a mean age of 24.32 ± 4.59 were evaluated. The patients received Iranian (group A) and Swiss (group B) riboflavin in 2 groups. The differences of all of the baseline indices were not statistically significant between the two groups.

The mean UCVA showed an improving trend in group A until the end of the 6^th^ month (P = 0.168) while in group B, it resumed its improving trend after a decrease in the third month (P = 0.577). The trend of the 6-month results did now a significant difference between the two groups (P = 0.239). The mean BCVA showed a decrease in both groups one month after the procedure but improved afterwards, although it was not significant. The trend of the BCVA changes was not significant between the two groups (P = 0.707).

After 6 months, MRSE decreased in group A but remained stable in group B. In general, the mean 6-month changes of MRSE was not significant between the two groups (P = 0.513). The trend of the changes of the above-mentioned indices is shown in Table 1.

**Table 1:** Comparison of the results between two groups of keratoconus patients receiving Iranian and Swiss riboflavin

				**After surgery**			P-value*
	**Riboflavin**	**No of eyes**	**Pre operation**	**1 months**	**3 months**	**6 months**	
UCVA (logMAR)	Sina Darou, Iran	30	0.82 ± 0.66	0.60 ± 0.52	0.52 ± 0.39	0.45 ± 0.36	0.239
	Uznach, Switzerland	30	0.82 ± 0.55	0.67 ± 0.54	0.79 ± 0.54	0.73 ± 0.51	
BCVA (logMAR)	Sina Darou, Iran	30	0.23 ± 0.28	0.25 ± 0.21	0.24 ± 0.19	0.19 ± 0.13	0.707
	Uznach, Switzerland	30	0.22 ± 0.18	0.27 ± 0.22	0.24 ± 0.19	0.20 ± 0.21	
Sphere (diopter)	Sina Darou, Iran	30	-1.53 ± 2.56	-1.70 ± 2.43	-1.80 ± 2.45	-1.42 ± 2.36	0.937
	Uznach, Switzerland	30	-1.59 ± 1.82	-1.64 ± 2.45	-1.57 ± 2.33	-1.63 ± 2.33	
Cylinder (diopter)	Sina Darou, Iran	30	-2.48 ± 1.77	-2.93 ± 2.07	-2.17 ± 2.17	-2.36 ± 1.79	0.242
	Uznach, Switzerland	30	-2.65 ± 1.94	-2.83 ± 1.88	-3.20 ± 2.04	-2.77 ± 1.93	
Spherical equivalent (diopter)	Sina Darou, Iran	30	-2.77 ± 2.51	-3.16 ± 3.02	-2.88 ± 3.13	-2.60 ± 2.85	0.513
	Uznach, Switzerland	30	-2.96 ± 2.28	-3.10 ± 2.78	-3.16 ± 2.75	-3.01 ± 2.70	

*Related to the comparison of the trend of the changes between the two groups using repeated measure ANOVA.

**Figure 2:**
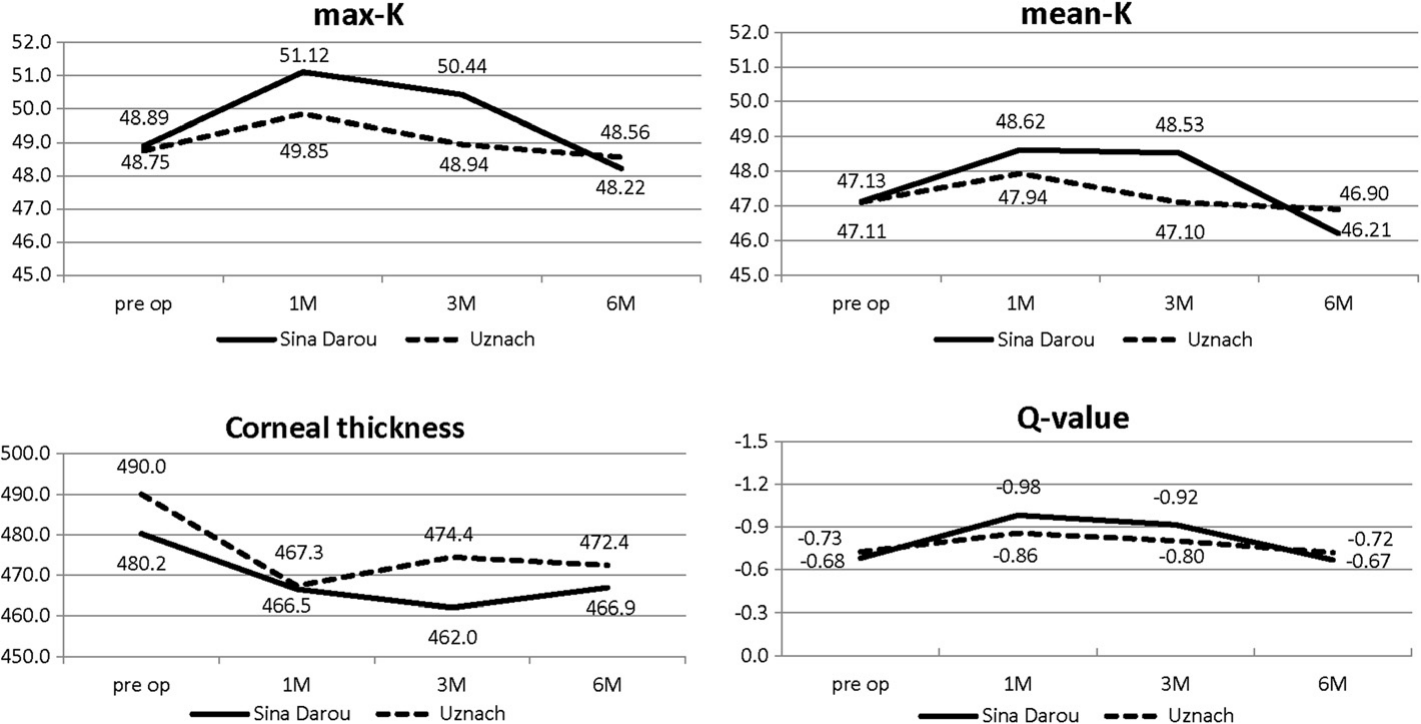
The temporal changes of max-K, mean-K, corneal thickness and Q-value between two groups of keratoconus patients receiving Iranian and Swiss riboflavin.

Despite an increase in max-K in the first follow up, it had a decreasing trend thereafter. The flattening was significant in neither group. In general, the decreasing trend of max-K was similar in both groups until the end of the sixth month (P = 0.731). Mean-K also showed the same decreasing trend with no significant difference between the two groups (P = 0.264). CCT showed a small insignificant decrease in both groups with no significant difference in 6 months (P = 0.759). The Q-value insignificantly shifted toward a prolate shape (more positive) in both groups but the 6-month trend of the changes showed no significant difference between the two groups (P = 0.669) (Figure 2).

Mean ± SD of corneal hysteresis (CH) was 7.70 ± 1.63 before the procedure in Group A which decreased to 6.76 ± 1.54 mmHg six months after the surgery (P = 0.008). In group B, CH decreased from 7.38 ± 2.10 to 7.23 ± 1.56 mmHg (P = 0.630). The decrease in CH between the two groups was borderline significant (P = 0.057).

Mean ± SD of corneal resistance factor (CRF) decreased from 7.02 ± 1.90 to 6.32 ± 1.30 mmHg in group A (P = 1.000) and from 6.80 ± 2.03 to 6.80 ± 1.84 mmHg in group B (P = 1.000); the difference between the two groups was not significant (P = 0.117).

The mean ± SD of ECC in group A was 2815.4 ± 243.1 and 2415.8 ± 317.8 cell/mm^2^ before and after the surgery, respectively (P < 0.001). In group B, ECC decreased from 2751.0 ± 261.2 to 2467.0 ± 255.9 cell/mm^2^ (P < 0.001). The decrease in ECC showed no significant difference between the two groups (P = 0.229).

## Discussion

CXL decreases or arrests the progression of keratoconus. After CXL, the corneal rigidity increases by up to 4.5 times [6] and the effects of the strengthening remain for a long time [2,3]. Although there are still concerns regarding regression due to the unknown collagen turnover time in the cornea [7,8], the 5-year results of treatment [3] can reduce the concern to a great extent. In this procedure, riboflavin produces free radicals under the effect of UV. These radicals create new covalent bonds in the stroma which strengthen the corneal tissue [1].

The role of riboflavin in this procedure is to increase UV absorbance and protection against its destructive effects on the cornea and lower layers. In vivo, riboflavin decreases the cytotoxic effects of UVA by up to 10 times [5]. On the other hand, it increases UV absorbance by up to 95% [4]. Without riboflavin, only 25-35% of UV is absorbed in the cornea [9] and reaches 50% in the lens which is undesirable [10]. This absorbance rate is influenced by riboflavin concentration. UV absorbance increases linearly in concentrations up to 0.04% while higher concentrations have no effect on absorbance [11].

CXL is considered a desirable treatment option and besides efficacy, it minimizes the cytotoxic effects on the epithelium with no cellular damage in the corneal endothelium and lens. During the procedure, epithelial cellular death occurs to a depth of approximately 300 micron; however, due to their high repopulation property, they regenerate after 6 months [12-14] with no dramatic effects on the treatment outcome [15,16]. On the other hand, the regeneration speed is very low in the endothelial cells and their damage is irreversible [11]. Clinical studies have shown that the cellular damage of CXL in the epithelium is within acceptable ranges and have reported no pathological damage from the endothelium to the retina after CXL [11,16,17]. Therefore, riboflavin is a mainstay of treatment.

Imported riboflavin is now used in Iran. Easier accessibility and lower costs persuaded us to use the Iranian riboflavin (Sina Darou). A preliminary study showed that both drugs were similar in formulation and active ingredient.

The clinical use of the drug in our study showed its efficacy, and the trend of the changes of vision, refraction, and topography was similar in both groups. In other words, although refraction, vision, and keratometry of the patients were deteriorating in the past year, development of the collagen bands arrested the process 6 months after the surgery with even improvement in some patients. The rate of the changes was similar in both groups, and also similar to a previous study we conducted using Swiss riboflavin [3]. Some studies reported that vision and refraction remained stable without significant changes [18] while some other studies reported a significant improvement in vision and refraction [19,20]. Of course, differences in the follow-up period, participants’ age, and study population may be the reasons for the different results.

In shorter follow-up, it should be kept in mind that the results of paraclinical tests may be affected by the corneal haze of CXL.

In our study, corneal keratometry changed similarly in both groups, showing that the process of protrusion and steepening stopped similarly in both treatment groups. Therefore, the inter- and intra-fibril bands were well developed in the group that received Iranian riboflavin. Wollensak et al. [1] reported that max-K decreased by 2.01 D in a follow-up period of 3 months to 4 years. In our study, the decrease was 0.6 and 0.3D in the Iranian and Swiss riboflavin groups, respectively. The reason for the difference could be different surgical methods; wollensak et al. removed the 7 mm central cornea while we removed a number of epithelial strips. Comparative studies should be designed and performed to evaluate this hypothesis.

CH, CRF, and ECC significantly decreased in the group that received Iranian riboflavin. Since the cytotoxic effects of the procedure on the epithelial cells continues for up to 6 months [12-14], the results of the procedure after one year or more should be used to evaluate CH, CRF, and ECC changes.

Of course, it should be noticed that despite laboratory reports of increased corneal rigidity following CXL, numerous clinical studies have found no change in the corneal biomechanical properties after CXL and different follow-ups have shown that the corneal biomechanical changes are stable without improvement [21-26].

## Conclusion

In general, it could be stated that vision, refraction, and corneal topography following CXL using Iranian (Sina Darou) and Swiss riboflavin are similar based on the 6-month results. However, considering the different decreases in CH, we require long-term studies to replace Swiss riboflavin with the Iranian one. Therefore, this study will continue to report the long-term results.

## Abbreviations

CXL: Collagen cross linking; MRSE: Manifest refraction spherical equivalent; BCVA: Best corrected visual acuity; UCVA: Uncorrected visual acuity; max K: Maximum keratometry; CCT: Central corneal thickness; ECC: Endothelial cell count; CH: Corneal hysteresis; CRF: Corneal resistance factor.

## Competing interests

The authors declare that they have no competing interests.

## Authors’ contributions

HH, MA, MM and HB have designed and supervised the project and advised on writing the paper. AS has performed primary study (laboratory study). SA analyzed the data and wrote the manuscript. HH, MA, MM and HB finalized the manuscript. All authors read and approved the final manuscript.
